# Decision-making styles in the context of colorectal cancer screening

**DOI:** 10.1186/s40359-020-0381-1

**Published:** 2020-02-03

**Authors:** Linda N. Douma, Ellen Uiters, Danielle R. M. Timmermans

**Affiliations:** 1 Department of Public and Occupational Health, Amsterdam Public Health research institute, VU University Medical Center, Van der Boechorststraat 7, 1081 BT Amsterdam, The Netherlands; 2 National Institute for Public Health and the Environment (RIVM), Postbus 1, 3720 BA Bilthoven, The Netherlands

**Keywords:** Colorectal cancer, CRC screening, Cancer screening, Decision-making, Decision-making styles

## Abstract

**Background:**

Our study examined the use of decision-making styles, as identified by Scott and Bruce (1995) (i.e. differentiating between a rational, intuitive, dependent, avoidant and spontaneous decision-making style), within the context of colorectal cancer (CRC) screening participation. In the field of cancer screening, informed decision-making is considered important, which follows the Rational Decision model. Subsequently, gaining more insight into decision-making styles being used in real life, could improve support to people when making their screening decision. In addition, we examined whether the decision-making style that people used was associated with their experienced decisional conflict.

**Methods:**

An online survey was carried out among a sample of first-time CRC screening invitees (1282 respondents, response rate 49%). We assessed people’s decision-making styles, CRC screening participation, education level, self-reported health literacy, and decisional conflict, and examined the possible associations between them.

**Results:**

In our study, people who had to decide about CRC screening scored high on using both a rational and intuitive decision-making style. Respondents scoring higher on using a spontaneous or dependent decision-making style were more likely to have participated in CRC screening, while respondents scoring higher on using an avoidant decision-making style were more likely *not* to have participated in CRC screening. However, differences were small. Generally, people in our study experienced low decisional conflict.

**Conclusion:**

Our eligible CRC screening population scored high on using both a rational and intuitive decision-making style. To optimise support to people, public education materials could be appealing more to the intuitive processes at hand. That being said, the current education materials aimed at informed/rational decision-making do not necessarily seem to create a problem, as people generally experienced low decisional conflict. Possible concerns regarding the use of a spontaneous, dependent or avoidant decision-making style could be that these styles might be contributing to less informed decisions. However, it is relevant to consider that the found differences are small and that any possible concern applies to a relatively small group of people.

## Background

Every day people are confronted with health-related decisions they have to make. This can vary from decisions concerning how to achieve a healthy lifestyle to decisions concerning the treatment of existing health conditions. The decision whether or not to undergo preventive colorectal cancer (CRC) screening is also a form of a health -related decision that people at some point in their life are confronted with, especially in developed countries such as the Netherlands. Preventive CRC screening is aimed at healthy individuals who are not experiencing any symptoms in order to detect cancer in an early stage or precursors of it. Its purpose is to reduce the number of cancer cases, treatments (invasive and non-invasive) and cancer deaths. Since January 2014, everyone in the Netherlands between the ages of 55 and 76 years old biennially receives an invitation to participate in CRC screening via a self-administered stool test (immunochemical faecal occult blood test: iFOBT), which is payed for by the government. People are expected to decide for themselves whether or not they want to participate in CRC screening. Thus, no one else other than the individual it concerns is actively involved in the decision-making process. If the stool test gives a positive result, people are referred for a colonoscopy to find out if they actually have (precursors of) colorectal cancer. In 2016, 72% of those invited for the first time to partake in the CRC screening programme in the Netherlands decided they wanted to be screened [1], which is relatively high compared to other countries.

Reducing the incidence and mortality of CRC are considered the main possible benefits of CRC screening [2–5]. However, CRC screening also involves possible downsides, such as false-negatives, false-positives, overdiagnosis, overtreatment, and risks associated with colonoscopy [6–10]. Especially on an individual level, it is not apparent whether the possible benefits outweigh the possible downsides of screening as different people could weigh these differently [8, 10, 11]. This has led to experts in the field of cancer screening presently considering it important that people make an informed decision concerning CRC screening participation [11–14]. The underlying framework for the concept of informed decision-making is the Rational Decision model [15, 16]. The Rational Decision model assumes that people base their decision on making maximum use of information and rationally weighing all aspects involved. Additionally, it assumes that people have stable preferences. Thus, following the Rational Decision model, it is increasingly believed to be important that people eligible for CRC screening have information available about CRC screening in order to make their own informed decision, thereby assuming that people will weigh the possible pros and cons of CRC screening against their personal values and preferences regarding screening [12, 17, 18]. However, this rational approach may not suit everyone confronted with this decision. Firstly, research on decision-making in general shows that different people can deal differently with the decisions in their life, which could be described as using different decision-making styles. The decision-making style that people use can be related to personality traits, for example, a ‘need-for-cognition’, which would promote a more informed and thoughtful process [19, 20], or a ‘need-for-closure’, which would promote a more speedy process [21, 22]. Additionally, people’s goals and motivation (regarding both their health and the accuracy of their decision) are involved. People who experience a personal relevance, will be held accountable, or have a reason to be accurate are more inclined to make an analytical and well-considered decision [23–26]. However, the decision-making style that people use can also be context-dependent and thus be associated with the specific situation or decision at hand. For example, a new or more complex decision often invokes a more thoughtful process, while time constraints can stimulate the use of intuition and heuristics as well as going off other people’s behaviour [23–25]. Furthermore, previous research on CRC screening shows that factors other than information are also associated with people’s decision to participate in CRC screening or not, such as beliefs, experiences, intuition, feelings, social norm, social support, and practical barriers [27–34]. Additionally, in a recent qualitative study we found CRC screening non-participants to prefer a more informed and rational process, while this appeared to be less so among CRC screening participants [35]. However, both CRC screening participants and non-participants mentioned that their decision involved a combination of reasoning and intuition or feeling. The findings of all this previous research combined suggest that those deciding about CRC screening participation could use decision-making styles other than, or in addition to, rational decision-making.

In the field of cognitive science, decision-making style is generally seen as “a habit-based propensity to react in a certain way in a specific decision context” [36]. There are different ways of conceptualising decision-making style, with many distinguishing between an intuitive and deliberative decision-making style [37], which is based on the dual-processing model regarding information processing [38]. However, Scott and Bruce’s (1995) General Decision Making Style (GDMS) inventory is the most comprehensive and widely validated conceptual approach [36, 39–43]. By conducting a large-scale project using four separate populations, Scott and Bruce developed a theoretically based measure of decision-making styles, resulting in the identification of five general decision-making styles: 1) a rational style, where the emphasis lies on a thorough and logical process; 2) an intuitive style, where the emphasis lies on the use of intuition and inner feelings; 3) a dependent style, where the emphasis lies on relying on the advice of others; 4) an avoidant style, where the emphasis lies on avoiding the actual decision-making; and 5) a spontaneous style, where the emphasis lies on making the decision as soon as possible and in the moment [36]. These decision-making styles are independent, but not mutually exclusive [36, 42]. It is possible that people use more than one of these five decision-making styles, but one can be preferred regarding a specific context. The decision-making styles identified by Scott and Bruce, especially the rational, intuitive and dependent style, seem applicable to the decision about CRC screening participation, because it involves dealing with information and weighing of the pros and cons of screening, but also people’s beliefs, experiences, intuition, feelings and social environment [12, 27–34]. However, to our knowledge, decision-making style in the context of CRC screening, or other cancer screenings, has received little attention so far, with the exception of Ghanouni et al. (2017) [44], who found (using GDMS) that having more of a rational decision-making style was associated with reading more of the cervical and CRC screening leaflets.

Assessing people’s decision-making styles used provides insight into their approach of making the decision. However, it does not reflect how people themselves evaluate the decision they made and the accompanying decision-making process. As previously described, the decision-making style that people use can be affected by both personal and context factors, which influence people may not always be actively aware of. Although the use of a certain decision-making style can be a ‘habit-based reaction’, that does not mean that people are always satisfied or confident with the style they used to make their decision, nor with the final decision made. People may feel that ideally they would have used a different approach. This possibility may particularly exist regarding people using a spontaneous or avoidant decision-making style, as previous research suggests that the use of these styles are associated with negative life outcomes (e.g. lower decision-satisfaction, less goal-achievement, worse mental health or more negative life events) [39, 43, 45–47]. Should people with these styles experience less satisfaction with their decision and decision-making process, then this is something to consider with regards to how CRC screening is currently being offered in the Netherlands, especially concerning the emphasis on people making their CRC screening decision by themselves without the involvement of any other party. Perhaps some people require more support in order to make their decision in a manner that they feel satisfied with. A common way to establish how people evaluate their decision and decision-making process is to assess people’s decisional conflict concerning their CRC screening decision [48]. People’s decisional conflict is thought to be affected by the extent to which they feel informed, how clear they are about their personal values, and how much they feel supported in or pressured into making a choice [48].

Examining which decision-making styles (following Scott and Bruce’s GDMS) are used when deciding about CRC screening participation, and the association with people’s evaluation of their decision, could contribute to the further development of education strategies and materials. The current education strategies and materials are aimed at fostering informed decision-making. However, people using a different decision-making style than a rational style might fare better with strategies and materials adjusted to their decision-making style (e.g. by providing different formats, appealing more to the role of intuition or other people, offering layered information (especially in combination with online facilities), or actively involving an expert/physician in the decision-making process) [18, 34]. The objective of this would be to improve support to people when making their screening decision and optimising their decision-making process. When examining the relationship between the decision-making styles used and people’s CRC screening participation and decisional conflict, it would also be relevant to adjust for possible associations with people’s education level and health literacy. Previous research on CRC screening participation has shown that people with a lower education or low health literacy have potentially more difficulty with processing complex and large amounts of information, which is often involved with the CRC screening decision [49, 50]. This could make them more inclined to rely on other factors in making their decision, possibly resulting in a preferred decision-making style other than a rational style. Additionally, people with a lower education or health literacy level are more likely not to participate in CRC screening [51, 52]. Thus, to sum up, with this study, we aimed to specifically address the following research questions:

1. When deciding about CRC screening participation, which decision-making styles (as described by Scott and Bruce) do people score high on using, and do they score highest on using a rational decision-making style?
2. Is there an association between people’s actual CRC screening participation and the use of a specific decision-making style? Hereby adjusting for possible associations with people’s education level and health literacy.
3. To what extent do people evaluate their CRC screening decision positively (in terms of decisional conflict), and is this associated with their CRC screening participation or the decision-making style they used? Hereby adjusting for possible associations with people’s education level and health literacy.

## Methods

### Recruitment of respondents

A full description of the recruitment approach used can be found in a previous publication [53]. A national online research panel (I&O Research Panel, www.ioresearch.nl, ISO 26362) was used to recruit people to participate in our questionnaire. In January 2014, a national preventive CRC screening programme has been introduced in the Netherlands, which involves biennially performing a self-administered stool test (immunochemical faecal occult blood test: iFOBT). People are eligible for CRC screening when aged between 55 and 76 years old. In 2014, only people from five birth years were invited to participate in CRC screening for the first time. Every year after that, during a period of 5 years, other birth years were gradually added to the invitation list. Eventually, in 2019, everyone eligible for CRC screening is invited for CRC screening. In 2016, the following birth years were expected to be invited for the first time for CRC screening: 1941 (age 75), 1945 (age 71), 1953 (age 63), 1955 (age 61), 1957 (age 59). These were the people we were interested in to take part in our study. This resulted in us inviting 2818 panel members from these birth years via e-mail in March 2017 to complete our online questionnaire.

### Questionnaire

Respondents were asked to fill out a questionnaire concerning their views on CRC screening, which also involved questions that are not part of the study being reported on in this article [53]. Relevant to the present study, respondents were first presented with the question whether they had participated in CRC screening or not (dichotomous variable: score 1 = participated, score 2 = did not participate), followed by the decisional conflict questions, and then the decision-making style questions. The health literacy questions were asked last.

### Measures

#### Decision-making style

To measure which decision-making styles people used within the context of CRC screening, we used Scott & Bruce’s General Decision Making Style (GDMS) questionnaire [36]. A Dutch version of the questionnaire was created using the back-translation method performed by two independent linguists (one Dutch native speaker and one UK native speaker). Care was taken to ensure that the questionnaire was understandable for people with at least B1 reading level. As previously described, the GDMS questionnaire differentiates between the use of five decision-making styles: 1) a rational style (*Cronbach’s α* = .68); 2) an intuitive style (*Cronbach’s α* = .82); 3) a dependent style (*Cronbach’s α* = .81); 4) an avoidant style (*Cronbach’s α* = .85); and 5) a spontaneous style (*Cronbach’s α* = .65). The measure contains 25 questions (e.g. ‘My decision-making requires careful thought’; ‘When I make decisions, I tend to rely on my intuition’), with five in each subscale, and all measured on a 5-point Likert scale ranging from strongly disagree (1) to strongly agree (5). Higher scores on each subscale (the sum of the items) mean that this style is used more frequently. See Additional file 1 for the complete questionnaire.

#### Decisional conflict

To measure how people evaluate their CRC screening decision in terms of decisional conflict, we used an existing Dutch version [54] of O’Connor’s Decisional Conflict Scale [48]. The Decisional Conflict Scale consists of 16 items (e.g. ‘I know the benefits of each option’; ‘I feel sure about what to choose’), which together are a measurement of decisional conflict (*Cronbach’s α* = .81). Each question is measured on a 5-point Likert scale ranging from strongly disagree (1) to strongly agree (5). The overall scale score is the mean of the sum of the items. A score of ‘1’ means people experience high decisional conflict and a score of ‘5’ means people experience low decisional conflict. See Additional file 1 for the complete questionnaire. As people in our sample had already made their decision concerning CRC screening participation, adjustments in wording were made accordingly when necessary (e.g. ‘I know the benefits’ became ‘I knew the benefits’).

#### Sociodemographic characteristics

Data on sex, age (birth year) and education (low, intermediate, high according to the International Standard Classification of Education (ISCED), 2011) were gathered.

#### Health literacy

To assess self-reported health literacy we asked respondents to answer the Set of three Brief Screening Questions (SBSQ) of Chew et al. 2008 [55] on a 5-point interval scale (1 = never/not at all, 5 = always/very): 1. How often do you have someone help you read hospital materials?; 2. How confident are you filling out medical forms by yourself?; 3.How often do you have problems learning about your medical condition because of difficulty understanding written information? A higher score on this Health Literacy Scale (the sum of the items; item 2 reverse-coded; *Cronbach’s alpha = .54*) means people have less adequate health literacy. Subsequently, people are usually categorised as having either adequate health literacy (sum score < 9) or inadequate health literacy (sum score ≥ 9) [55, 56].

#### Statistical analysis

We analysed the proportion of people scoring 15 or higher on the use of a specific decision-making style (15 being the midpoint). This was solely done for descriptive purposes as knowing how large the group is that scores relatively high on using a specific decision-making style can be useful for screening practice. All subsequent analyses are based on the original variables and range of scores (5–25). Correlations between the decision-making scales were computed. All decision-making styles are independent variables. Using multiple logistic regression analysis, we first examined the possible association between each decision-making style independently/separately and CRC screening participation in order to get a first estimate of the association between each one of the independent variable and the outcome measure CRC screening participation, hereby adjusting for possible confounding by education and self-reported health literacy [57]. Subsequently, using multiple logistic regression analysis, we examined the associations between the decision-making styles and CRC screening participation while also adjusting for the found interrelatedness between the decision-making styles (i.e. all decision-making styles were entered together into one regression model). Using multiple linear regression analysis, the same approach was used to examine the possible associations between the decision-making styles and decisional conflict. Multiple logistic regression analysis was used to examine the possible association between CRC screening participation and decisional conflict. As described above, Chew’s health literacy measure is commonly dichotomised, categorising people into having either adequate or inadequate health literacy [55, 56]. However, because our sample did not have enough participants with inadequate health literacy (*N* = 20) to conduct analyses with self-reported health literacy entered as a dichotomous variable, we entered self-reported health literacy as a continuous variable. All analyses were carried out using IBM SPSS Statistics 24.0.0.1.

## Results

### Sample characteristics

The sample characteristics are summarized in Table 1. A full description can be found in a previous publication [53]. People with a higher education were overrepresented in our sample and the majority of our study sample (98%) had adequate health literacy. Additionally, only 11% of our sample were CRC screening non-participants, compared to 28% of the actual CRC screening population in the Netherlands in 2016 [1]. Non-response analysis showed that higher educated people were more likely to have participated in our survey. Regarding sex or birth year, no significant differences were found.

**Table 1:** Characteristics research sample

Variables	*N* (%)
Total sample	1282 (100)
Screening participation	
Yes	1142 (89)
No	140 (11)
Sex	
Male	773 (60)
Female	509 (40)
Education	
Low	258 (20)
Intermediate	404 (32)
High	611 (48)
Birth year	
1941	127 (10)
1945	228 (18)
1953	329 (26)
1955	297 (23)
1957	301 (23)
Health literacy level	
Adequate health literacy (sum score below 9)	1262 (98)

### Decision-making styles

Different decision-making styles were used, with on average people scoring the highest on having used a rational and intuitive style and the lowest on having used an avoidant style (Table 2). We found several significant correlations between the different decision-making styles (Table 3). The strongest correlations were between intuitive style and spontaneous style (*r* = .47, *p* < .001) and between dependent style and avoidant style (*r* = .50, *p* < .001).

**Table 2:** Descriptive statistics regarding the different decision-making styles, Decisional Conflict Scale and Health Literacy Scale^a^

Variable	Possible range	Actual Range	M (SD)	Median	Mode	% scoring 15 (scale midpoint) or higher on decision-making style	Skewness
Rational decision-making style	5–25	5–25	18.32 (2.71)	19	20	86	−.56
Intuitive decision-making style	5–25	5–25	17.72 (3.50)	18	20	75	−.38
Dependent decision-making style	5–25	5–25	13.11 (3.74)	13	14	26	−.07
Avoidant decision-making style	5–25	5–24	9.91 (3.39)	10	10	6	.65
Spontaneous decision-making style	5–25	5–25	13.79 (2.97)	14	13	28	.13
Decisional Conflict Scale	1–5	2.25–5	4.10 (.47)	4.12	4	–	−.50
Health Literacy Scale	3–15	3–12	4.73 (1.61)	4	3	–	.84

^a^ *N* = 1282

**Table 3:** Correlation matrix of decision-making styles

Decision-making style	Intuitive style	Dependent style	Avoidant style	Spontaneous style
Rational style	−.06*	.25**	−.05	−.20**
Intuitive style		.01	.02	.47**
Dependent style			.50**	−.04
Avoidant style				.05

* *p* < .05

** *p* < .001

### Association between decision-making style and CRC screening participation

When examining the possible association between each decision-making style separately and CRC screening participation (Table 4 – A), the spontaneous and avoidant decision-making styles were significantly associated with CRC screening participation. When examining the associations between the decision-making styles and CRC screening participation while also adjusting for the interrelatedness between the decision-making styles (i.e. all decision-making styles were entered together into one regression model; Table 4 – B), the dependent decision-making style was also significantly associated with CRC screening participation. People who scored higher on using a spontaneous or dependent decision-making style were more likely to have participated in CRC screening. People who scored higher on using an avoidant decision-making style were more likely not to have participated in CRC screening. No significant associations were found between the rational, intuitive, and dependent decision-making styles and CRC screening participation.

**Table 4:** Associations between: A. Decision-making styles and CRC screening participation, with each decision-making style entered separately in a multiple logistic regression model; B. Decision-making styles and CRC screening participation, with all decision-making styles entered together into one multiple logistic regression model^a^

A. Each decision-making style separately – CRC screening participation	OR^b^	95% CI
Rational decision-making style – CRC screening participation	.996	.932–1.064
Intuitive decision-making style – CRC screening participation	.967	.918–1.019
Dependent decision-making style – CRC screening participation	.992	.946–1.041
Avoidant decision-making style – CRC screening participation	1.074*	1.021–1.129
Spontaneous decision-making style – CRC screening participation	.928*	.873–.987
B. All decision-making styles together in one model – CRC screening participation	OR^c^	95% CI
Rational decision-making style – CRC screening participation	1.004	.935–1.078
Intuitive decision-making style – CRC screening participation	.997	.940–1.058
Dependent decision-making style – CRC screening participation	.940*	.886–.997
Avoidant decision-making style – CRC screening participation	1.111**	1.047–1.178
Spontaneous decision-making style – CRC screening participation	.924*	.861–.991

^a^ Association models, with CRC screening participation entered as dependent variable (score 1 = participation, score 2 = non participation). Decision-making styles are the independent variables; higher scores mean the style is used more frequently (scores range from 5 to 25)

^b^ Rational style: OR adjusted for education and self-reported HL, significant confounding found regarding both variables

Intuitive style: OR adjusted for education and self-reported HL, significant confounding found regarding education.

Dependent style: OR adjusted for education and self-reported HL, significant confounding found regarding self-reported HL.

Avoidant style: OR adjusted for education and self-reported HL, significant confounding found regarding both variables.

Spontaneous style: OR adjusted for education and self-reported HL, no significant confounding found regarding both variables.

^c^Rational style: OR adjusted for education and self-reported HL, significant confounding found regarding both variables

Intuitive style: OR adjusted for education and self-reported HL, significant confounding found regarding both variables.

Dependent style: OR adjusted for education and self-reported HL, no significant confounding found regarding both variables.

Avoidant style: OR adjusted for education and self-reported HL, no significant confounding found regarding both variables.

Spontaneous style: OR adjusted for education and self-reported HL, no significant confounding found regarding both variables.

*Significant at *p* < .05

**Significant at *p* < .001

### Decisional conflict regarding CRC screening decision

Generally, people experienced low decisional conflict (which is represented by a higher score on the decisional conflict scale) regarding their CRC screening decision (Table 2). However, there was a significant difference associated with participation, with those experiencing less decisional conflict being more likely to have participated in CRC screening (Table 5 – A).

**Table 5:** Associations between: A. Decisional conflict and CRC screening participation (multiple logistic regression)^a^; B. Decision-making styles and decisional conflict, with each decision-making style entered separately in a multiple linear regression model; C. Decision-making styles and decisional conflict, with all decision-making styles entered together into one multiple linear regression model^b^

A. Decisional conflict – CRC screening participation	OR^c^	95% CI
Decisional conflict – CRC screening participation	.193**	.132–.282
B. Each decision-making style separately – Decisional conflict	B^d^	95% CI
Rational decision-making style – Decisional conflict	.045**	.036–.054
Intuitive decision-making style – Decisional conflict	.017**	.009–.025
Dependent decision-making style – Decisional conflict	−.001	−.008–.006
Avoidant decision-making style – Decisional conflict	−.043**	−.050 – -.036
Spontaneous decision-making style – Decisional conflict	.003	−.006–.011
C. All decision-making styles together in one model – Decisional conflict	B^d^	95% CI
Rational decision-making style – Decisional conflict	.042**	.033–.051
Intuitive decision-making style – Decisional conflict	.014**	.007–.022
Dependent decision-making style – Decisional conflict	.012*	.004–.019
Avoidant decision-making style – Decisional conflict	−.050**	−.058 – -.042
Spontaneous decision-making style – Decisional conflict	.004	−.005–.013

^a^ Association model, with CRC screening participation entered as dependent variable. Decisional conflict as independent variable; a higher score means less experienced decisional conflict (scores range from 1 to 5)

^b^ Association models, with decisional conflict entered as dependent variable. Decision-making styles are the independent variables; higher scores mean the style is used more frequently (scores range from 5 to 25)

^c^ OR adjusted for education and self-reported HL, no significant confounding found regarding both variables

^d^ Concerning all styles: betas adjusted for education and self-reported HL, no significant confounding found regarding both variables

* Significant at *p* < .05

** Significant at *p* < .001

### Association between decision-making style and decisional conflict

When examining the possible association between each decision-making style separately and decisional conflict (Table 5 – B), three of the five decision-making styles were significantly associated with experienced decisional conflict, with the exception of the dependent and spontaneous decision-making styles. When examining the associations between the decision-making styles and decisional conflict while also adjusting for the interrelatedness between the decision-making styles (i.e. all decision-making styles were entered together into one regression model; Table 5 – C), the dependent decision-making style was also significantly associated with experienced decisional conflict. People who scored higher on using a rational, intuitive or dependent decision-making style generally experienced less decisional conflict. People who scored higher on using an avoidant decision-making style, however, experienced more decisional conflict.

## Discussion

Our study examined the use of decision-making styles, as identified by Scott and Bruce (1995) [36] (i.e. differentiating between a rational, intuitive, dependent, avoidant and spontaneous decision-making style), within the context of CRC screening participation. Our results showed that most people in our study used a combination of decision-making styles, with scoring high and more or less equally high on using both a rational and intuitive decision-making style. Furthermore, we found that several decision-making styles showed a significant association with screening participation; respondents scoring higher on using a spontaneous or dependent decision-making style were more likely to have participated in CRC screening, while respondents scoring higher on using an avoidant decision-making style were more likely not to have participated in CRC screening. In addition, we examined the relationship between the use of decision-making styles and experienced decisional conflict. Generally, people in our study experienced low decisional conflict regarding their CRC screening decision. People who had not participated in CRC screening and people scoring higher on using an avoidant decision-making style experienced relatively more decisional conflict.

Our finding that people scored high and highest on using the rational and intuitive decision-making styles is in agreement with what Ghanouni et al. (2017) [44] found in their study into factors affecting the reading of cancer screening information, as well as with findings from other studies on other health-related and non-health-related choice contexts [36, 39, 43, 58, 59]. Additionally, we found that people scoring higher on using a spontaneous or dependent decision-making style were more likely to have participated in CRC screening, while people scoring higher on using an avoidant decision-making style were more likely not to have participated in CRC screening. However, the differences are small, as are the proportions of people in our study scoring relatively high (i.e. 15 or higher on the subscale) on using a spontaneous (28%), dependent (26%) or avoidant decision-making style (6%). Thus, the effect is not very strong and especially from a screening practice perspective, it is relevant to consider that any possible concern related to this finding applies to a relatively small group of people. Nonetheless, previous research on decision-making styles suggests that, generally, a rational or intuitive decision-making style is associated with more positive decision outcomes (e.g. higher decision-satisfaction, more goal-achievement, better mental health or less negative life events); while particularly an avoidant or spontaneous decision-making style is associated with more negative decision outcomes [39, 43, 45–47]. Regarding CRC screening participation, there is no single objective ‘right’ decision to be made by an individual as one’s personal values and situation determines whether they believe the possible benefits of CRC screening to outweigh its possible downsides [11, 34, 60]. However, in view of informed decision-making [12, 14], it would be relevant to consider possible concerns regarding the decision-making style that people are using in the context of making an informed decision about CRC screening participation. People scoring higher on using a spontaneous decision-making style are more inclined to make their decision on the spur of the moment and feel a desire to make their decision as quickly as possible [36]. Supported by the negative association between the rational and spontaneous decision-making styles, this might mean that people using a spontaneous decision-making style decide to participate in CRC screening without thorough consideration of the pros and cons and without being sufficiently informed [40, 43]. Ghanouni et al. (2017) also provides support for this possibility as they found that people using a rational decision-making style compared to other styles had read a greater proportion of the bowel cancer-screening leaflet [44]. Regarding the use of a dependent decision-making style, a concern could be that people may be relatively blindly (i.e. uninformed) following the advice or example of others or feel pressured into participating [61]. The concern regarding people scoring higher on using an avoidant decision-making style being more likely not to have participated in CRC screening mostly involves whether they made their decision not to participate consciously and were well-informed [62]. However, in our present study, we focused on assessing whether or not people used a rational decision-making style, and whether the use of a specific decision-making style was associated with participating in CRC screening or not. We did not assess people’s actual knowledge concerning CRC and CRC screening, and thus their extent of making an informed decision about CRC screening, and whether this might be related to the use of a particular decision-making style. Therefore, further research is needed to establish whether using a spontaneous, dependent or avoidant decision-making style is actually a concern for making informed and well-considered CRC screening decisions.

In general, independent of CRC screening participation or decision-making style, people in our study experienced low decisional conflict, indicating that they felt certain about their CRC screening decision and positive about its quality. However, although again a small difference, people who had not participated in CRC screening were more likely to experience relatively more decisional conflict than people who had participated in CRC screening. This could be due in part to CRC screening participants receiving a (often positive) test-result – while CRC screening non-participants do not receive a test-result – and interpreting this as a confirmation of the correctness of their decision [60, 63]. It could also be related to using an avoidant decision-making style, which was more likely among CRC screening non-participants, as people scoring higher on using this decision-making style experienced relatively more decisional conflict. It is possible that people with an avoidant decision-making style feel more uncertain in general, or that their experienced decisional conflict stems from having avoided making the decision [58, 64], perhaps especially in combination with the behaviour of not participating. Future research could shed more light on underlying mechanisms (including possible mediation effects). The positive association between having an avoidant decision-making style and experiencing decisional conflict underpins the relevance of assessing the extent to which people displaying the use of this decision-making style consciously decided not to participate in CRC screening.

Our study also examined the association patterns between decision-making styles, which were in agreement with previous findings concerning GDMS [36, 39, 43, 58]. Previous studies on GDMS have shown inconsistent results regarding the relationship between the rational and intuitive decision-making styles. In our study, we found a negative association between the rational and intuitive decision-making styles, although very small and marginally significant. Additionally, we found a negative association between the rational and spontaneous decision-making styles, and a positive association between the intuitive and spontaneous decision-making styles. This seems logical as people with a spontaneous decision-making style are likely not to have or to take the time to think about all the options and information available as extensively as people with a rational decision-making style do, and were able to benefit from using their feelings and intuition as an instant source of information [36, 40, 43, 65]. In addition, we found a positive association between the rational and dependent decision-making styles. It could be that in the context of CRC screening people seek information and advice from others as an active part of their rational decision-making strategy, or in order to reduce effort [59, 66]. Furthermore, we found a positive association between the dependent and avoidant decision-making styles. This could be an indication of people who avoid making the CRC screening decision tending to rely more on others for advice or for making the decision itself [40, 58, 64].

Discussing the association patterns we found, contributes to the existing body of knowledge regarding research using the GDMS. However, more importantly, it shows the complexity of decision-making regarding CRC screening in real life. It confirms that the use of any decision-making style takes place within a context of the existence of other styles. Additionally, it gives insight into whether certain groups or types of people might be distinguished, which subsequently offers insight into barriers as well as opportunities for how to best reach people and support them in their decision-making process. For example, the correlation between the rational and dependent decision-making styles gives rise to the opportunity to also stimulate rational/informed decision-making by addressing it as part of a dependent decision-making style (e.g. by asking relevant others for advice). Additionally, the correlation between the avoidant and dependent decision-making styles offers support for the potential that, for a subgroup of people, incorporating a third party in the decision-making process, such as a general practitioner, which is currently not yet the situation in the Netherlands, could have a positive effect.

In our study, we first examined the possible association between each decision-making style independently/separately and CRC screening participation, followed by an analysis where we adjusted for the found interrelatedness between the decision-making styles (i.e. all decision-making styles were entered together into one regression model). When adjusting for the interrelatedness between decision-making styles, people scoring higher on using a dependent decision-making style were more likely to have participated in CRC screening (*OR* = .940, *95% CI:* .886–.997, *p* = .04), while without this adjustment a non-significant association was found (*OR* = .992). A similar result was visible regarding the association between decision-making styles and decisional conflict (a significant association with the dependent decision-making style was only found when adjusting for the interrelatedness between decision-making styles). How to interpret this finding is not directly evident as the dependent decision-making style has a positive correlation with the rational decision-making style, which when independently analysed showed an association with CRC screening participation, as well as with the avoidant decision-making style, which when independently analysed showed an association with CRC screening non-participation. Future research could further examine the complex interplay between decision-making styles in the context of CRC screening.

Our study has several limitations. First, the participants in our study were members of a national internet panel. As it is possible that they are different from people who do not involve themselves with online research, this could limit generalizability. Generalizability may also be affected by the fact that our sample included a relatively small proportion of CRC screening non-participants (11%), compared to the 28% of those invited for the first time to partake in CRC screening in the Netherlands in 2016 deciding not to participate [1]. Also considering that, CRC screening uptake in the Netherlands is already relatively high compared to other countries. Additionally, our sample included a relatively large proportion of higher educated people (48%) as well as people with adequate (self-reported) health literacy (98%), which is thus not representative of the general population. Furthermore, it should be noted that regarding the analysis of health literacy we used a continuous variable instead of a dichotomous variable, the latter being the more common way to identify adequate versus inadequate health literacy [55, 56]. Caution should also be taken in interpreting our health literacy results, as reliability analysis showed low internal consistency (Cronbach’s alpha is .54). A final limitation is the fact that we measured decisional conflict retrospectively, which is not uncommon regarding this concept. However, for a proportion of our sample this can mean that people were asked about their decisional conflict a year after they made their decision to participate in CRC screening or not. This could have resulted in people feeling more confident about their decision partly because of more time having passed since they made their decision.

## Conclusion

In our study, the eligible CRC screening population scored high on using both a rational and intuitive decision-making style when deciding about CRC screening participation. Thus, the strong emphasis on making an informed CRC screening decision, which follows the Rational Decision model, does not appear to be congruent with decision-making in real life. To optimise support to people when making their CRC screening decision, public education materials could be appealing more to the intuitive processes at hand. That being said, from a personal evaluation perspective, the current education materials that are attuned to a rational decision-making style do not necessarily seem to create a problem, as people generally experienced low decisional conflict, independent of decision-making style, indicating that they felt certain about their CRC screening decision and positive about its quality. In our study, people scoring higher on using a spontaneous or dependent decision-making style were more likely to have participated in CRC screening, while people scoring higher on using an avoidant decision-making style were more likely not to have participated in CRC screening. Possible concerns in view of informed decision-making could be that these decision-making styles might be contributing to less informed decisions. However, it is relevant to consider that the found differences are small and that any possible concern related to this finding applies to a relatively small group of people.

## Supplementary information

**Additional file 1.** Questionnaires.

## Data Availability

The data supporting the conclusions of this article are included within the article. The datasets used and/or analysed during the current study are available from the corresponding author on reasonable request.

## Abbreviations

CRC: Colorectal cancer screening
GDMS: General Decision Making Styles
ISCED: International Standard Classification of Education
ISO: International Organisation for Standardization
