# Supplementary material for: Decision-making styles in the context of colorectal cancer screening
Source: BMC Psychol. 2020 Feb 3;8:11. doi: 10.1186/s40359-020-0381-1 (PMC6998095; doi:10.1186/s40359-020-0381-1)
Supplement: Supplementary file 1 — Additional file 1. Questionnaires. [file 40359_2020_381_MOESM1_ESM.docx]

**Appendix A - Questionnaires**

1. **Decisional conflict scale (O’Connor, 1989)**

*Complete scale*

All 16 items

*Subscales/domains*

I = Informed

V = Values clarity

S = Support

U = Uncertainty

E = Effective decision

*Scoring*

1 = Strongly disagree

2 = Disagree

3 = Neutral (neither agree nor disagree)

4 = Agree

5 = Strongly agree

***Statements***

1) I know which options are available to me (I)

2) I know the benefits of each option (I)

3) I know the risks and side effects of each option (I)

4) I am clear about which benefits matter most to me (V)

5) I am clear about which risks and side effects matter most to me (V)

6) I am clear about which is more important to me (the benefits or the risks and side effects) (V)

7) I have enough support from others to make a choice (S)

8) I am choosing without pressure from others (S)

9) I have enough advice and information to make a choice (S)

10) I am clear about the best choice for me (U)

11) I feel sure about what to choose (U)

12) This decision is easy for me to make (U)

13) I feel I have made an informed choice (E)

14) My decision shows what is important to me (E)

15) I expect to stick with my decision (E)

16) I am satisfied with my decision (E)

1. **General Decision-Making Style (GDMS) questionnaire (Scott & Bruce, 1995)**

*Five styles*

R = Rational

I = Intuitive

D = Dependent

A = Avoidant

S = Spontaneous

*Scoring*

1 = Strongly disagree

2 = Disagree

3 = Neutral (neither agree nor disagree)

4 = Agree

5 = Strongly agree

***Statements***

1) When I make decisions, I tend to rely on my intuition (I)

2) I rarely make important decisions without consulting other people (D)

3) When I make a decision, it is more important for me to feel the decision is right than to have a rational reason for it (I)

4) I double-check my information sources to be sure I have the right facts before making decisions (R)

5) I use the advice of other people in making my important decisions (D)

6) I put off making decisions because thinking about them makes me uneasy (A)

7) I make decisions in a logical and systematic way (R)

8) When making decisions, I do what feels natural at that moment (S)

9) I generally make snap decisions (S)

10) I like to have someone steer me in the right direction when I am faced with important decisions (D)

11) My decision-making requires careful thought (R)

12) When making a decision, I trust my inner feelings and reactions (I)

13) When making a decision, I consider various options in terms of a specified goal (R)

14) I avoid making important decisions until the pressure is on (A)

15) I often make impulsive decisions (S)

16) When making decisions, I rely upon my instincts (I)

17) I generally make decisions that feel right to me (I)

18) I often need the assistance of other people when making important decisions (D)

19) I postpone decision-making whenever possible (A)

20) I often make decisions on the spur of the moment (S)

21) I often put off making important decisions (A)

22) If I have the support of others, it is easier for me to make important decisions (D)

23) I generally make important decisions at the last minute (A)

24) I make quick decisions (S)

25) I explore all of my options before making a decision (R)
